# Kinetics of Mononuclear Cell Subpopulations in the Peripheral Blood of Patients with Giant Cell Arteritis During the Acute Phase of the Disease: The Role of Steroids

**DOI:** 10.31138/mjr.33.1.102

**Published:** 2022-03-31

**Authors:** Ourania D. Argyropoulou, Dimitrios Anastasios Palamidas, Nikolaos Paschalidis, Paschalis Sideras, Athanasios G. Tzioufas

**Affiliations:** 1 Department of Pathophysiology, School of Medicine, National and Kapodistrian University of Athens, Greece,; 2 Biomedical Research Foundation Academy of Athens, Centre for Clinical, Experimental Surgery and Translational Research, Athens, Greece

**Keywords:** vasculitis, giant cell arteritis, glucocorticosteroids, CyTOF

## Abstract

**Background/Aim:**

Giant cell arteritis (GCA) represents the most prevalent form of systemic vasculitis in the elderly, primarily affecting the temporal artery, the extracranial branches of carotid arteries, and the aorta. GCA is a highly heterogeneous disease in terms of clinical and histological findings, pathophysiology, and treatment selection strategies. The disease is highly responsive to glucocorticosteroids (GCs), but almost half of patients may relapse following GCs tapering. The main hypothesis of GCA pathogenesis includes altered immune responses and changes in the vascular microenvironment, leading to a dynamic interplay between innate and adaptive immunity. The aim of this study is to explore the effect of GCs on the phenotype of peripheral mononuclear cell subpopulations and on the major inflammatory molecules detected in the peripheral blood of patients during the acute phase of the disease.

**Methods:**

Patient PBMCs will be studied using Cytometry by time of flight (CyTOF). Following the CyTOF analysis, Luminex Assay will be performed on the same patient samples to identify the kinetics of the most prominent inflammatory mediators correlating with the subpopulations detected. Patient population consists of 8 patients with GCA, 6 with polymyalgia rheumatica, as disease control group and 5 healthy controls (sex and age matched) at 3 time points: disease diagnosis, 48 and 96 hours after treatment administration.

**Conclusion:**

The identification of potential alterations in cell subpopulations and the kinetics of inflammatory mediators are expected to lead to the production of new knowledge regarding the role of corticosteroids in the phase of acute inflammatory response.

## BACKGROUND / INTRODUCTION

Giant Cell Arthritis (GCA) is the most common form of systemic vasculitis in people over 50 years of age. It is characterised by transmembrane granulomatous inflammation mainly of the middle and large elastic arteries. Its predominant feature is the multi-level heterogeneity: clinical (eg, systemic inflammation, cranial symptoms or generalized vascular disease affecting the aorta and its main branches, rheumatic polymyalgia [RP] [40–60%] and overlapping phenotypes) histological, pathogenetic and therapeutic.^1–4^ It should be underlined that GCA and Takayasu Arteritis exhibit great differences in both clinical, imaging and histopathology aspects making them two distinct clinical entities.^5^

Irreversible vision loss, ischemic stroke, and aortic aneurysms with or without rupture are the most serious complications in the course of the disease which require immediate treatment.^6–7^ Glucocorticosteroids (GCs) are the treatment of choice with immediate clinical response within 48–96 hours of onset. However, the high recurrence rate of the disease treated with GCs (34–62%),^8^ the pre-existing comorbidities of these patients and the complications of their long-term use, make it necessary to introduce new treatment strategies in daily clinical practice. Disease-modifying drugs such as methotrexate^9^ have been suggested, and the most effective, to date, biological treatment is the monoclonal antibody against the interleukin-6 receptor.^10^

The pathogenesis of GCA is characterised by modified immune responses and changes in the vascular microenvironment, in genetically predisposed individuals, which lead to a continuous, dynamic interaction between innate and adaptive immunity. Uncontrolled induction of abnormal maturation and proliferation of vascular dendritic cells (DCs) induces a strong T-cell response resulting, initially, in the infiltration of diseased tissue by CD8 + and mainly CD4 + T-lymphocytes. Subsequently, the latter, under the influence of pro-inflammatory factors, differentiate, mainly into T-helper-1 (Th1) and Th17 cells, leading to uncontrolled stimulation and migration of monocytes and macrophages to the site of inflammation, but also B-lymphocytes. Fibroblast expansion is an immediate consequence of extensive vascular inflammation, contributing to vascular remodelling. All the above pathophysiological processes are mediated by pro-inflammatory and regulatory cytokines, angiogenic and growth factors such as: IL-12, IL-18, IL-23, IL-1β, IL-6, IL-17A, IL-21, IL-32, IL-33, TNF-α, TGF-β, IFN-γ, VEGF, PDGF, FGF and ET-1.^11^ B lymphocytes also appear to contribute to the modulation of T-cell responses through the secretion of pro-inflammatory and / or anti-inflammatory cytokines.^12^ The increased expression of several interleukins, both in serum and in temporal artery biopsies (TABs) of patients with GCA, have been suggested as a possible biomarker of prognosis and response to treatment.

Strong IL-17A expression has been associated with fewer relapses and the need for shorter-term treatment.^13^ In contrast, increased IL-12 expression has been associated with more severe and extensive disease and cranial complications, while IL-23 correlates with multiple flares.^14^ In patients with active GCA and PMR, elevated serum IL-6 levels may be associated with a stronger inflammatory response, frequent relapses, and the need of higher doses and longer GC treatment.^15–17^

Among the different cell populations involved in the pathogenesis of GCA are neutrophils, as suggested by the presence of different neutrophil phenotypes, both in the peripheral blood and in the inflamed vessels of these patients.^18–22^ Recently, our team described, for the first time in the literature, their ability to release neutrophil extracellular traps (NETs) into the inflamed temporal artery tissue, which are decorated with the pro-inflammatory cytokines IL-6 and IL-17A which play a pivotal role in the pathogenesis of the disease,^23^ possibly contributing to the vicious cycle of induction and/or perpetuation of the inflammatory process. Changes in different cell populations after initiation of GCs therapy in both GCA and PMR patients have been studied sporadically.

More specifically, studies noted: a) a reduction in only non-classical monocytes in patients with GCA, while all the monocytic subpopulations were reduced after 3 months of treatment with GCs in the PMR group,^24^ b) a reduction in peripheral B cell numbers in patients with active GCA and PMR compared to healthy controls and a rapid subpopulation recovery, as well as their ability to produce IL-6 after 2–12 weeks of treatment,^12^ c) 3 distinct neutrophil phenotypes at 48 hours, 1 and 24 weeks after treatment initiation with progressive increase, by phenotype, of endothelial adhesion capacity, and overproduction of CXCL5, IL-8, IL-17, and IL-6 at 24 weeks.^21^

The dynamic state of immune cell subpopulations in the peripheral blood of patients with GCA and PMR and the simultaneous study of the differential expression of disease relevant inflammatory mediators in the patient serum during the phase of rapid clinical improvement have not been studied to date.

## AIM OF THE STUDY

The proposed study aims to investigate the effect of GCs on distinct cell subpopulations of peripheral mononuclear cells (PBMCs) in patients with GCA, using mass cytometry (Cytometry by time of flight; CyTOF) and Luminex Assay. The results of this study may reveal new cell subpopulations and/or molecules that could be proved either useful biomarkers for monitoring disease outcome and response to treatment, or more importantly new therapeutic targets.

## RESEARCH PLAN - METHODS

The proposed study aims to investigate the effect of GCs on distinct cell subpopulations of peripheral mononuclear cells (PBMCs) in patients with GCA, using mass cytometry (Cytometry by time of flight; CyTOF) by specific antibodies which allows the simultaneous and multi-level characterisation of different cell subpopulations in the same sample. For this purpose, the following populations will be studied: a) whole T cells and their main subpopulations, such as active CD4 + and CD8+ cells (naive and memory) and their subtypes (Th1, Th2, Th17 helper, and T regulatory cells) and natural killer T cells (NKT), b) whole B cells and their major subpopulations, such as mature B cells, plasma cells and memory B cells, c) monocytes / macrophages (M1 and M2), dendritic cells and their subtypes, such as myeloid and plasma cell dendritic cells, and (e) natural killer cells (NK; early and mature). The changes in the above populations will be characterized at three different time points that cover the phase of immediate therapeutic response of patients: time of diagnosis (0 hours), 48 and 96 hours after the initiation of GCs treatment.

Depending on the cell subpopulations detected and the type of immunity observed, we will proceed with the selection of a Luminex Assay panel of angiogenic and growth factors and cytokines to study the differential expression of major inflammatory mediators. This experiment will be performed on serum samples of patients included in the CyTOF analysis for all three time points mentioned above.

The study population includes: a) patients with GCA (n = 8), b) patients with PMR (n = 6) as a disease control group, and c) healthy controls (matched by age and sex, n = 5). The criteria for inclusion in the study are: age over 18 years, absence of recent diagnosis of infectious disease (at least 1 month before the diagnosis) and absence of underlying immunodeficiency or neoplasia (at least 5 years before the diagnosis of GCA and/or PMR), while all patients must meet the relevant ACR/EULAR classification criteria per disease. 60% of the samples have already been collected and stored at −80°C in the biobank of the Laboratory of Pathophysiology, until the analysis is performed.

All patients are monitored in the external rheumatology clinic of Pathophysiology from 02/2020 until today, while the laboratory and imaging data during the diagnosis of the disease of all patients will be collected from the medical records and will be studied retrospectively. The mass cytometry analysis, as well as the Luminex Assay technique will be performed in collaboration with the CyTOF Laboratory at the Institute of Biological Research of the Academy of Athens (IIBEAA). Primary experiments to optimize PBMC staining, and mass cytometry analysis have already been performed. The research protocol has been approved by the Committee of Bioethics and Ethics of the Medical School, NKUA (Protocol No.: 1718016656). All samples are collected with the written consent of the participants and in full compliance with GDPR rules.

The study will be developed in two stages and is expected to last a total of two years. During the first phase (lasting about 1 year) the collection of biological material will be completed, then mass cytometry (CyTOF) will be performed and the results will be analysed. In the second stage, after selecting the panel of molecules, the Luminex Assay will take place and will follow the final combined analysis of all data (duration about 1 year). The proposed study is expected to lead to the production of at least one publication and one poster presentation in an international conference as well as the Panhellenic Rheumatology Conference.

## IMPACT OF THE STUDY

The great progress that has been made in the last decades in the field of systemic vasculitis, including GCA, is overshadowed by unanswered questions and important gaps which concern: i) the phenotypic separation of patients, ii) the finding of new diagnostic, prognostic and response to treatment biomarkers, iii) the identification of new therapeutic targets, and iv) the application of personalised medicine, both in the management and treatment of these patients, the benefits of which concern primarily the patient, and secondly, their family and the National Health System. All the above can be achieved through a better understanding of the disease’s pathogenetic mechanisms involved in the perpetuation of the inflammatory response. The main players of inflammation are the immune cell populations that orchestrate the innate and adaptive immunity by acting directly or indirectly through the production of pro-inflammatory and / or anti-inflammatory molecules, angiogenic and growth factors. The shifts in cell populations, as well as the molecules that they produce during the phase of rapid reversal of inflammation, under the action of GCs, have not been studied to date. Their identification, which is the aim of the proposed study, is expected to lead to the production of new knowledge about the role of GCs in the phase of acute inflammatory response and on the other hand to potentially reveal new cells and/or target molecules capable of predicting the outcome of the disease and more importantly to guide the therapeutic choice by revealing new therapeutic goals.
